# “It Stays with You for Life”: The Everyday Nature and Impact of Police Violence in Toronto’s Inner-City

**DOI:** 10.3390/ijerph191710503

**Published:** 2022-08-23

**Authors:** Carolyn Greene, Marta-Marika Urbanik, Kanika Samuels-Wortley

**Affiliations:** 1 Faculty of Humanities and Social Sciences, Athabasca University, Athabasca, AB T9S 3A3, Canada; 2 Department of Sociology, University of Alberta, Edmonton, AB T6G 2H4, Canada; 3 Department of Criminology, Toronto Metropolitan University, Toronto, ON M5B 2K3, Canada

**Keywords:** police violence, health inequity, mental health, trauma, public health

## Abstract

In recent years, police violence has amassed notable international attention from the public, practitioners, and academics alike. This paper explores experiences and perceptions of police violence in Canada, documenting the impacts of direct and vicarious experiences of police violence on inner-city residents. The study employed semi-structured interviews with 45 community members across three Toronto inner-city neighbourhoods. Using a general interview prompt guide, participants were asked a range of questions about their experiences with and perceptions of police, and particularly, of police violence in their community. The interviews were audio recorded, transcribed, thematically coded, and analyzed. All participants reported direct and/or vicarious experiences of police violence, and most described experiencing long-standing, and continual fear that police contact would result in harm to them. Further, participants described a variety of serious and negative outcomes associated with experiencing and/or witnessing police violence. Police violence in Canada is a public health issue that requires an integrated public health policy approach to address the negative outcomes associated with direct and vicarious police violence exposure.

## 1. Introduction

Concerns over police violence have re-emerged to the fore of public consciousness following numerous high-profile police murders of unarmed Black and Indigenous men and women in the United States and Canada. Instances of lethal police violence captured on video have initiated broad and sustained media attention and sparked growing recognition of the broader public health implications of police violence. Although discussions of police violence have recently entered the mainstream, little has been done to address the intersecting structural inequalities of race and poverty that create and reinforce disproportionate levels of invasive and lethal policing in racialized, low-income neighbourhoods across North America.

Research demonstrates that the risk of experiencing police violence is heightened for those living in territorially stigmatized and socioeconomically disadvantaged areas [1,2]. Research has also found that neighbourhood racial composition is a more significant predictor of the nature and frequency of police encounters than poverty [3]. As a result, marginalized and racialized persons—specifically, Black and Indigenous persons—continue to face disproportionate police contacts and criminalization across the United States [4,5] and Canada [6,7,8] thereby increasing their likelihood of harmful police interactions, including lethal force [9,10].

International studies have documented the deleterious effects of police violence on various public and social health outcomes. Specifically, researchers have found direct and indirect exposure to police violence is highly traumatic and contributes to increased risk of mental health problems, including stress, hypervigilance, depression, anxiety, and post-traumatic stress disorder [11,12,13,14,15,16,17,18,19]. Such experiences and related collateral consequences increase morbidity [11,12,13,15,18,19,20] and adverse physical health outcomes (e.g., obesity, high blood pressure) [20,21,22,23] and contribute to poorer educational outcomes (e.g., lower high school grades and completion rates) [24,25]. Yet, rich empirical documentation and analysis of the individual and collective impacts of discriminatory and violent police actions remain limited within the Canadian literature in public health.

Media discourse in Canada often frames police violence as atypical and a less pressing problem than in the USA, often citing comparatively low incidence rates [1,26]. Such framing minimizes the prevalence and consequences of police violence in Canada. Critically, this framing also neglects the disproportionate risk and impacts of police violence on certain groups; compared to all other groups, Black and Indigenous persons in Canada are more likely to be subjected to police violence—and specifically, police use of lethal force [27,28,29,30]. To illustrate, between 2000 and 2017, Black persons represented 37% of the victims of police use of deadly force in Toronto while comprising just 8.3% of the population. In Winnipeg, Indigenous persons accounted for about 66% of victims of police lethal force while comprising only 10.6% of the population [31].

Given the long history of police violence in Canada, it is curious that there is so little research on its public health impacts. One notable exception is the research examining the experiences and perceptions of police treatment among people who use drugs (PWUD) in Canada. Scholars have documented significant primary and secondary public health impacts associated with adversarial policing, which includes the impact on PWUD’s access to harm reduction/healthcare services and the secondary effects (or consequences) that lack of service access can have on individual health and wellbeing [32,33,34,35,36]. These studies reveal that how the police treat PWUD can have critical public health implications. Yet, beyond the public health implications of police harassment and violence documented among this population, we continue to know little about the health impacts of police violence on people living in/spending time in marginalized neighbourhoods in Canada. Thus, although some Canadian work has provided rich accounts of the hardships inner-city residents are subject to, including over-policing and neighbourhood violence, there is little empirical data on the individual and community level effects of police violence [37].

To address this paucity of research, this study explores how individuals experience and understand the impacts of police violence across three Canadian inner-city communities. To account for the range of experiences participants described, we apply the World Health Organization’s definition of violence, which includes psychological, physical, sexual, and neglectful [20] Findings reveal that participants perceived and experienced police violence as pervasive, inescapable, and deeply traumatizing. Informed by Mitton [38] and Decker et al. [39], we argue police violence in Canada is a public health crisis that requires: (1) recognition of macro level (structural) factors (e.g., structural/systemic racism, lack of police accountability, poverty) that contribute to police violence within low-income, oft racialized communities; and, (2) an integrated public health policy approach informed by both macro and micro (individual) level factors (e.g., anxiety, post-traumatic stress) implicated in the negative health and educational outcomes associated with exposure to police violence. Our paper aims to shift discourse away from more narrowly focused work on the roles of law/policy and individual police officers/citizens with respect to police violence, and toward a broader understanding of the health impacts associated with direct and indirect exposure to police violence [40].

## 2. Materials and Methods

The data for this paper were collected as part of a larger study that explored how early and repeated police contacts throughout a person’s lifetime impacts perceptions of police and policing among people connected to three Toronto inner-city neighbourhoods: Regent Park, St. Jamestown, and Blake-Boultbee. These areas hold a high density of social housing (for additional neighbourhood description see [41], and when compared to other, more affluent areas of Toronto, such social housing projects are often burdened by disproportionate levels of gun violence.

This paper is based upon 45 semi-structured, non-directive interviews and 12 follow-up interviews conducted between 2016 and 2021. Participants were located and initially recruited through existing social and professional networks previously established by the researchers. Specifically, Urbanik’s 6 years of ethnographic research in the Regent Park and Greene’s long-standing personal relationships in the neighbourhoods. We then relied upon non-probability, snowball sampling to extend our participant base. Prior to the COVID-19 pandemic, we spent considerable time in these communities, which was possible given the researchers previously established networks. It is well established that participant referrals, especially for hard-to-reach populations, require pre-existing relationships to be successful [42]. Although previous contact with police was required, given the type of fieldwork carried out, all participants reported some type of police contact, with many participants reporting multiple police encounters as either victims, suspects, or simply as neighbourhood residents. Of this sample (n = 45), 73.3% of participants identified as Black (25 male; 8 female), 8.9% as Indigenous (1 male; 3 female), 11.1% as white (1 male; 4 female), and 2.2% as Asian (1 male). All participants had completed some of high school; however, few participants had received a high school diploma. Two participants had completed college.

Participants were interviewed in various locations across the study neighbourhoods, including in parks, on front porches, in cars, and in peoples’ homes. The interviews were audio-recorded, transcribed, anonymized, coded, and ranged in length from 20 min to several hours (1 h average). Given our participants’ lived realities, our relationships with residents were important as many feared police retaliation for sharing their experiences [43]. Such relationships also allowed us to triangulate our findings where possible. It is important to note that participants described their experiences with police within a broader net of victimization attributable, in part, to aggressive, pro-active policing within their neighbourhoods. Experiences and perceptions of police harassment, physical assault, theft, misconduct, humiliation, and coercion were commonly shared by all participants regardless of their current/past levels of criminal involvement. Compared to women, men reported experiencing more frequent and more severe physical violence in their encounters with police, though women were not immune to police violence.

Data analysis was an ongoing and iterative process throughout data collection. We employed deductive and inductive thematic analysis to ensure the data steered the identification and development of recurring themes [44]. We pursued themes as they arose during interviews until we reached thematic saturation [45,46]. We immersed ourselves in the data at each stage of data analysis, re-reading interview transcripts multiple times to identify initial free-codes [47]. Utilizing an “open-coding” process, we initially identified 6 main/“organizational” codes (e.g., perceptions of police, experiences with police). Further analysis led to the identification of 17 sub-theme/“substantive” codes (e.g., experiences of police physical violence, trauma) based on the organizational codes (pp. 107–108 [48]).

To ensure organizational and substantive codes accurately represented our data and participants’ views, Authors Urbanik and Greene and two research assistants independently identified organizational and substantive codes for five interviews. Inter-coder reliability checks resulted in 90% agreement on all organizational/substantive codes. Following Braun and Clarke’s [44] best practices in qualitative analysis, we reviewed transcripts again ensuring that data were captured sufficiently in relation to codes, and that the participant quotations chosen to represent the organizational/substantive codes were representative of the data.

## 3. Results

Participants reported long histories of police contact beginning in early adolescence. All participants reported witnessing police violence (n = 45) and 89% (n = 40) reported direct experiences of police violence (specifically, physical violence). Participants’ accounts of police violence were deeply traumatic. Incidents of violence spanned across time—years prior to just days before their interview—and place, such as walking through their neighbourhoods, being in their homes, and/or being en route to or in a police station. Finally, although police violence was reported by diverse racial/ethnic groups across these low-income neighbourhoods, Black and Indigenous participants reported more severe violence, including graphic accounts of serious physical and verbal (often racist) assaults by police. Further, although men reported experiencing police violence more frequently than women, some women—particularly Indigenous women—also reported violent police encounters.

### 3.1. The Prevalence and Pervasiveness of Police Violence

Participants believed police violence in Toronto was prevalent, Ashton explains: “*Honestly, in Ontario police brutality, not just Toronto, but mainly Toronto …is at an all-time high in the history of Canada*” (35 years). Participants often nonchalantly described verbal and physical abuse by police, framing these as part of “*everyday life*”. In these communities, police violence fell within the context of individual and community expectations of police behaviour, and as such, police violence was normalized and expected. Kalia (28 years) states: “*It’s normal to get beat up by cops, that’s normal to everyone …It’s NOT ‘Oh my god, I can’t believe that happened!’ It’s a joke of, ‘How bad did they beat you?’ Guys would joke about which cop it was*”. Jacob (29 years), in recalling a police officer threatening to shoot him in the face, similarly describes the normalcy ascribed to police violence: “*it was just normal because that’s what happens down there, and it happened to me more than once.*”

Participants described violent police encounters starting in their early teenage years or when police no longer viewed them as young children, as Trent explains: “*When you’re 16, 17, you may have facial hair, you could get a beating at that point because they’re not sure you’re a child*”. Further, participants reported experiencing/witnessing increasingly severe violence over time: “*It went from ‘Whatever-you got hit with a Billy stick a couple times’ to like ‘I think they’re gonna kill him’*” (Kalia). Further, incidents of police violence were precipitated by both voluntary and involuntary police contacts. Participant accounts of involuntary police encounters resulting in violence often entailed police stops in the neighbourhood. Jacob recounted his first violent police encounter at 14, when officers, suspecting drugs were being sold, approached his friend group at an outdoor birthday celebration:

“*The police officer …standing in front of me was a white officer and he really got mad. He’s like ‘What you think I’m fucking stupid? Come here,’ grabs me by my throat, picked me off of bike and choked slammed me right to the ground …and he finally grabbed me by my hair, picked me up and threw me on the ground face first and cuffed me. When he was cuffing me his partner comes over. He had his knee in my back …I’m like ‘I can’t breathe’. [Police]‘Shut up you fucking n—r’ and all these guys are watching me. I don’t know what to say, we were all young, right?*”

Jacob’s interview took place 4 years prior to the police murder of George Floyd in 2020, making his “*I can’t breathe*” statement particularly unsettling. Jacob’s experience was just one of many accounts of police violence that he, and other participants, reported experiencing directly and/or witnessing others experience in the respective communities.

Although participants’ frequently discussed police violence within the context of involuntary contacts (street stops), some participants also reported violent victimization during voluntary interactions (e.g., calls for service/medical assistance). Many participants described the outcomes of such interactions negatively. To illustrate, Megan (22 years) describes how after calling police for assistance, she tried to intervene when police “roughed up” her brother:

“*My sister ran back downstairs, she was like ‘Oh, come, like they’re roughing up Omar!’, and I came upstairs and we tried recording them. They were like ‘Stand back! Stand back! Don’t talk! Don’t talk! There’s police involved!’ I’m like ‘What the fuck-I’m the one who called you guys to come bring my brother upstairs! They ended up fucking restraining me, throwing me down on the fucking floor, ripping my hair out. They were literally pulling my fucking hair, and …then they ended up charging me for obstruction.*”

Megan reported being deeply angered and traumatized by this encounter. Consider also Eli’s (18 years) experience. Eli reported bleeding out in an apartment hallway after being stabbed, when a neighbour called 911. He recounted an officer stepping on his chest wounds as he lay on the ground, struggling to breathe. The officer then told him he ‘*hoped [he] died before paramedics arrived*’. As Eli elucidates, “*I’ve been victimized by police more than I’ve been victimized by anybody in my life.* *I’ve had a cop step on me while I was dying and scared, stepped on my chest, like I’ve had a cop literally try to let me die before*”. As a consequence of such experiences, many participants did not believe they could rely on police during times of need.

While participants described police violence as common, many participants perceived police violence was most likely to occur when they were not engaging in criminal activity. That is, participants felt when police stopped them or their friends and did not find any ‘evidence’ of wrongdoing, this frustrated officers and increased the likelihood police would react violently, as Dave explains (28 years): “*Once they see you looking at them [police] they jump out the car …AND when they don’t find anything?! That’s the next beating! They slap you up*”. Based on Dave’s experience, walking through one’s own neighbourhood whilst innocent [not in possession of contraband], could trigger “*the next beating*”. Equally troubling, police violence also occurred when participants reported complying with officers’ demands. Chris (42 years) recounts being assaulted by police after questioning why he was stopped while walking home from school: “*They didn’t find nothing on me, but because I was lippy to them, I got some hits and some kicks …he kicked me in my ass. Sent me on my way. But that’s regular stuff man, that’s not even something to complain about, really*”. It is notable Chris explained the assault as a consequence of being “lippy to” police, by asking why they stopped him. When Chris was asked about this, he clarified that in this incident and all other police encounters: “*I was polite to be honest. I would ask ‘what are you stopping me for?’ or ‘what did I do?’ Fear stopped me from showing how I really felt. I figured the fastest way out of those situations was not to agitate*”. Participants’ accounts not only reveal the frequency of police abuses, but they also highlight the fear these encounters can evoke.

### 3.2. The Impact of Police Violence

#### 3.2.1. Fear and Its Effects

Most participants believed violent police encounters were inescapable. This caused pervasive fear of police interactions. For example, Liam (23 years) described his thought process when coming across officers while walking through an alleyway: “*I was …by myself, so I was so scared. Oh my god, I’m gonna end up like Trayvon Martin*”. He then clarified how fear placed him and other Black youth at greater risk of police suspicion and harm: *“…They’re so afraid that they may say something and it appear …like they’re trying to hide something. ‘Oh, you look nervous, you’re trying to hide something’. No! You’re just scaring the shit out of them, they can’t even say their name”. Given this fear, many participants weighed the risks of interacting with police: “Maybe I should run? Are they gonna beat me if I stick around? …If I stay, they might say, “Hey guess what? We got to take you to the station’ …Meanwhile it’s like, ‘Why are you taking me?’”* (Liam). This fear also produced anxiety about interacting with police: “*I don’t usually get anxiety, but I get anxiety that I don’t want them to come question me. I don’t want them to come talk to me. I don’t want them to come near me!*” (Clayton-38 years).

Participants also expressed fear for their loved ones’ safety, and some described how these fears directly impacted their educational trajectories. Jacob recalled when, at 16, he entered his home to find police officers assaulting his mother. He shared his anger about what happened and how fear of this happening again led to his dropping out of school:

“*I was young …and scared to be honest …I was also angry. I felt like to pick them up and throw them over the balcony at that age, at that time. Instead these officers spoke to me. And I really don’t want to hear their voice! I sat there in shock listening. And they said ‘just convince your mom to come to the hospital’ … So I convinced …her to go and …I had to leave her there. And that’s why I dropped out of school because I was scared it might happen again, you know?*”.

Jacob’s experience highlights the profoundly negative impacts of witnessing a loved one assaulted by police, and it illustrates the hidden impacts of police violence in shaping educational outcomes for those who experience and/or witness it.

Participants also expressed immense fear of reporting police violence. Monica (48 years) describes why she did not report the racist verbal assault she was subjected to after police entered her home: “*I just thought that I didn’t want to get further on the wrong side [of police]. I didn’t want our family, my home, to become a target, you know? And having seen the backlash against other women who complained, I just didn’t think it was worth the risk*”. Brice (34 years) described the police “*jumping on*” him and his friend when they incorrectly suspected them of drug trafficking, breaking his friend’s bones. When Brice went to the police station to report the incident, he said to the neighbour accompanying him: “*‘What do you think is going to happen when I go inside?’ I go, ‘Watch this’. I tried to report …and the fucking guy [police officer] ripped the paper in front of me! Ever since then, I say fuck these guys*”. Participants commonly described being discouraged from formally reporting their victimization. Zaidy (48 years) recalled an assault where police “*kicked me and stomped on me, in my head, in my back, in my legs and stuff*” and officers threatening that “*if I reported, they were gonna be able to charge me for assaulting them, and that they’d beat me up more*”. Kristal (23 years) similarly described wanting to lodge a complaint and a lawyer discouraging her because it “*wouldn’t go far*”, making her feel helpless: “*if you want to tell on them, you can’t even tell on them*”. Kalia recounted wanting to sue police over her treatment during a lengthy criminal investigation: “*They traumatized me more than anyone in the world. I fear them so much! I want to sue them so bad, but I’m scared, I’m so scared to file a complaint. They’re gonna harass you over that. You’re crazy to think that you could make a complaint! They [police] tell it to us all the time*”. The emotional turmoil victims of and witnesses to police violence felt was palpable. Although many participants wanted police to be held accountable, their fears of retribution prevented most from reporting. This context likely explains why, after being mistreated and subject to police violence, only 2 participants lodged formal complaints.

While participants almost uniformly believed they would become a police “target” (Chris) if they filed formal complaints, they also believed their experiences would be doubted, particularly when pitted against police officers’ accounts. To illustrate, when asked if they had ever considered filing a complaint about police violence, participants often responded with disbelief and amusement at the question: “*What is that going to do?! [laughing]*” (Marcus-39 years) “*Who is gonna listen to you, really? Who is gonna believe you?...Nobody! I’ll give your head a slap, shut up!*” (laughing) (Mira-37 years). For participants, neighbourhood outsiders “…*turned a blind eye*” (Ty 24 years) to their experiences because of the structural inequalities—particularly poverty—they faced. “*What’s the point? They’re not gonna believe someone from Regent Park over a cop*”. Terrill (29 years) explains why his family did not take their concerns to authorities following a family member’s death while in police custody: “*They’re so economically strapped…and probably very afraid, you know? …if the had money, they [police] would be in some fucking serious trouble*”. Thus, despite research showing most people indicate a willingness to report victimization by police [49], our participants felt their experiences would be doubted and dismissed, and feared they would place themselves at further risk of future victimization via “harassment”, being “beat up more”, and/or police illegitimately charging them if they filed formal complaints.

#### 3.2.2. Effects on Mental Health

Many participants reported mental health trauma resulting from direct and vicarious experiences of police violence, and unsurprisingly, older participants—those with the most frequent/severe exposure—reported this most often. Kalia explains: “I wasn’t subjected to it [physical assault], but I would see it. And it’s hard to see someone in handcuffs and get stomped on. It was a hard thing to watch; the more things I saw them doing, it started traumatizing me”. To address the trauma caused by police violence, Terill shared: “I write songs that express my hate and opposition for them [police]. That shows how deep the traumatization is…I should keep that to myself, but it’s SO deep, other people have to know”. Indeed, the trauma participants described was longstanding. For example, CJ (39 years) recounted his best friend, Mac (14 then) and another child, Dante (11 then) playing in a building hallway when a police officer approached them, threatened Dante, and put a gun into his mouth. CJ shared becoming preoccupied with this incident and describes Mac’s ongoing trauma, “Every time this guy is drunk…he calls me, [saying] ‘I remember when they [police] did that to Dante, man. Remember the cop had the gun in his mouth?’ Still to this fucking day and…[Mac] is 45 [now]…to this day he still remember that shit”. When asked if this story had impacted him, CJ responded: “Of course! I’ll never forget that shit”. Although over twenty years have passed, this encounter continues to have a lasting impact on those who experienced it directly and those—like CJ—who merely heard about the incident.

Although only two participants indicated being officially diagnosed with a mental health disorder, many others described symptoms consistent with trauma-related health concerns. Chris, who has led a ‘prosocial’ life for over a decade now, described his feelings when seeing the police: “I still have phobia [of police]. When I see a police cruiser, I look the opposite direction. It stays with you for life …you get traumatized by it”. Almost all participants expressed the “phobia” of police that remains “for life”, even as participants moved out of the respective neighbourhoods. Alex (44 years) explains the longstanding physiological and emotional impact of seeing police: “I could feel it in my stomach. I don’t want them to see me, talk to me. Now if they comin closer, fuck it, it’s like, flight or fight. Cuz you know they can fuck you up!” Participants’ accounts highlight how police violence, as well as the fear it produces, likely contributes to heightened stress and trauma-related symptomology that is going undiagnosed among some participants. This finding also likely reflects the existing research documenting the many significant challenges racialized persons experience in accessing appropriate mental healthcare services in Canada [50,51,52].

Many participants reported behaviours and mental health symptoms consistent with depression, anxiety, and (complex) post-traumatic stress disorder (e.g., sweaty palms, racing heart, nightmares, avoidance) as a result of police interactions, even when police were not around. Chris, further articulating his feelings, described: “*I get depression. Sometimes I just look at things [experiences with police] and say why me? You know? I know I did some things when I was young, but what do they* [police] *expect when they treat you like that?...the fucked-up thing is they* [police] *still come for you* [as a pro-social adult]”. Chris recognizes that police violence has negatively impacted his mental health, however, he has never been diagnosed, has never sought help and critically, indicated never having been offered counselling—despite frequent child welfare, education, and justice system interventions during his youth. Another participant, Alex (45 years) explains: “*Sometimes I don’t even wanna leave my house. And even then I can’t get away from them, cuz sometimes I even have fucking dreams about that shit! I know it’s something wrong with me*”. Of the two participants that reported formal diagnoses, one shared: “*I have anxiety and depression. When I got older, I figured that out. One of my kids has it too. I know the system so we got a bit of help. We would probably have got nothing if I didn’t*” (Kate, 45). The other expressed: “*My doctor declared me Post Traumatic Stress, and I decided let them think I’m crazy or whatever. Just fuck it*” (Lana-38 years).

CHECK INTERVIEW ID INFO DEMO

## 4. Discussion

Participants in this study reported extensive histories of police violence and threats, to the point where they expected police violence. Despite this normalization, participants recounted the profoundly negative consequences of these direct and/or indirect experiences. Our findings suggest that the trauma associated with direct/indirect experiences of police violence in Canada is likely contributing to a range of negative health outcomes which warrant greater research attention and public health response. Participants’ descriptions of police violence were situated within a broader net of police victimization experiences, such as harassment, theft, humiliation, and coercion, that are attributable, in part, to aggressive, pro-active policing strategies in their communities. As our data reveal, the combined exposure—through direct/indirect experiences—to “large and small events” had a cumulative effect on residents’ well-being. Further, although many participants alluded to mental health issues in discussing the trauma of police violence, few had accessed/received any mental health care. Importantly, recent research suggests that police violence may uniquely and independently (from other forms of co-occurring trauma and violence) affect physical and mental health [53]. Given this, it is likely that people impacted by police violence in Canada are experiencing significant unmet mental health care needs [54], and this may be particularly true for Black and Indigenous youth and adults who are disproportionately subjected to police violence.

Beyond the individual-level impacts of witnessing/experiencing police violence, structural inequality shaped participants’ experiences and outcomes. Black and Indigenous participants described disproportionate exposure to and more severe/frequent instances of police violence when compared to white participants. Participants that experienced the most frequent/severe police violence also described the most intense emotional impacts of police violence. This suggests Black and Indigenous inner-city residents may have even greater unmet healthcare needs than previously identified, which can exacerbate the existing heath inequities these community members face. Further, Black and Indigenous participants also reported being negatively affected by the added violence of racial discrimination (e.g., police using racial slurs). Certainly, police violence impacts the health of individuals and communities. When public discourse overlooks and/or minimizes these impacts, and when police officers are not held accountable for perpetrating violence, it communicates that victims’ “…bodies are police property, disposable, and underserving of dignity and justice” (p. 663, [11]). Said differently, when police violence persists as a result of the dominant society’s indifference and/or lack of action, it signifies the societal worth of community members, and ultimately suggests that the lives and well-being of Black and Indigenous persons do not matter [55]. Our findings further suggest that Black and Indigenous individuals in Canada are likely at far greater risk of negative health outcomes, as a result of the cumulative impact of the traumas resulting from both racial discrimination and police violence. Although this study provides a detailed exploration of residents’ accounts of experiences and views of police violence, it is limited to one Canadian city, and although snow-ball sampling was employed to diversify the sample within neighbourhoods, participants’ views may not be representative nor generalizable to other neighbourhood residents. We encourage future research to examine the impact of police violence across different locations and to identify the direct and indirect mechanisms by which police violence impacts individuals’ well-being, including long-term health and educational outcomes in Canada.

In addition to the significant mental and physical health effects of police violence, it is also important to note the implications of police violence on public safety, as it can damage community–police relationships. Unsurprisingly, intensive over-policing has resulted in racialized persons—particularly, Black and Indigenous persons—being more likely to fear police and view them negatively—often perceiving police as untrustworthy, racist, and dangerous [29,56], and this contributes to an unwillingness to cooperate with and even contact police [57]. When community members witness, experience, and otherwise hear about police violence and other forms of misconduct, this increases fear, decimates trust, and erodes perceptions of police legitimacy. These negative views then suppress willingness to report crime/cooperate in criminal investigations, and even contact police when help is needed for personal or public safety [57]. Such perceptions have vast implications for public health. First, when people are too afraid to call police for help, this increases the risk of victimization and even death [58,59]. Second, when people are unwilling to cooperate with police, this limits crime detection, control, and prevention. Although research has long documented the negative effects of fear of crime on peoples’ well-being, participants in this study reported extensive fear of police, the very people tasked with protection and security. As such, this fear must be understood as profoundly impacting community members’ well-being [60].

Importantly, it must be recognized that a public health approach alone will not end police violence. To address this public health crisis, a multifaceted approach that involves strategic—and where possible collaborative—actions from across sectors, including, but not limited to government, health, education, and police organizations is necessary to holistically address police violence [37]. The individual and collective trauma resulting from police violence requires both: (a) macro-level policy development that can address the structural inequalities that influence the conditions which allow police violence to persist, such as racial discrimination; and (b) the micro-level policies and practices that leave victims with unmet healthcare needs and impede them from seeking redress, such as police officer accountability, education for teachers and healthcare providers, reducing mental health stigma as it relates to police violence, and increasing access to mental health services. We encourage implementation and ongoing evaluation of interventions aimed at reducing police violence and its negative impacts.

## 5. Conclusions

This study supports the need for a deeper understanding of the individual and collective impacts of direct/indirect experiences of police violence currently and over one’s lifetime in Canada. The public health implications of police violence on individuals and communities are many, and although a public health approach alone will not end police violence, it can provide an important, and much needed shift, in how police violence is researched and responded to in Canada. Police violence, like other forms of violence, must be understood as a public health issue with wide ranging negative health implications.

## Data Availability

The data are not publicly available due to ethical concerns regarding the anonymity and confidentiality of participants. Data may be available upon request to Drs. Urbanik and Greene.
